# Association of admission C-reactive protein levels and histological chorioamnionitis in patients with preterm premature rupture of membranes: a systematic review and meta-analysis

**DOI:** 10.3389/frph.2026.1912720

**Published:** 2026-09-28

**Authors:** Cunling Zhang, Zhuo Wei, Dan Wu, Meiyi Xu, Wen Li

**Affiliations:** 1 Tianjin Institute of Gynecology Obstetrics, Tianjin Central Hospital of Gynecology Obstetrics, Tianjin, China; 2 Tianjin Key Laboratory of Human Development and Reproductive Regulation, Tianjin Central Hospital of Obstetrics Gynecology, Tianjin, China

**Keywords:** crp, HCA, meta-analysis, PPROM, systematic review

## Abstract

**Background:**

Preterm premature rupture of membranes (PPROM) is a high-risk obstetric complication. Ascending infection and histological chorioamnionitis (HCA) frequently occur after membrane rupture, contributing to severe adverse maternal and fetal outcomes. Early prediction of HCA using non-invasive biomarkers allows timely clinical management, while the predictive efficacy of C-reactive protein (CRP) for subsequent HCA in PPROM patients remains inconsistent across existing studies. This meta-analysis aimed to systematically evaluate the predictive association.

**Methods:**

We systematically searched PubMed, Scopus, Web of Science and the Cochrane Library from inception to May 20, 2026 for studies on serum CRP and subsequent HCA in PPROM patients. Pooled mean differences (MD) of admission CRP concentrations were calculated using random-effects meta-analysis with results visualized via forest plots. Leave-one-out sensitivity analysis confirmed robustness. Subgroup analyses and meta-regression were applied to explore potential sources of heterogeneity. Funnel plots, combined with Egger's test and Begg's test, were used to evaluate publication bias. All statistical analyses were conducted using R software (version 4.6.0).

**Results:**

Initially, 11 studies were screened. After excluding one low-quality study, 10 studies (1,791 participants) were ultimately included. The pooled results demonstrated that PPROM patients who subsequently developed HCA exhibited higher admission CRP levels than non-HCA controls (MD = 0.439, 95% CI: 0.179–0.700), with substantial heterogeneity (*I*^2^ = 87.7%). Meta-regression and subgroup exploration suggested sample size may partially drive cross-study heterogeneity; this moderator accounted for 31.19% of total variance, with a borderline significant association (*P* = 0.05). Sensitivity analysis confirmed the stability of the pooled estimates. Egger's test revealed no significant publication bias (*P* > 0.05), but the substantial heterogeneity warrants caution in interpretation.

**Conclusion:**

This meta-analysis suggests that elevated admission CRP levels are modestly correlated with subsequent HCA in PPROM populations. CRP may serve as an auxiliary indicator to assist early risk stratification; however, its clinical utility is limited by the small effect size and substantial heterogeneity across studies. Given the observational design of the included studies, these findings represent association rather than causation. Prospective cohort studies with standardized protocols are needed to confirm these results and assess the incremental value of CRP over existing clinical predictors.

**Systematic Review Registration:**

https://www.crd.york.ac.uk/PROSPERO/, identifier CRD420261404654.

## 1 Introduction

Preterm premature rupture of membranes (PPROM), defined as spontaneous rupture of fetal membranes before 37 weeks of gestation prior to the onset of labor, complicates approximately 2%–4% of all pregnancies and accounts for nearly one-third of preterm deliveries worldwide (1, 2). PPROM predisposes both mother and fetus to substantial infectious morbidity, including chorioamnionitis, neonatal sepsis, and adverse long-term neurodevelopmental outcomes (3). Intra-amniotic infection and inflammation represent the central pathophysiological process driving these adverse outcomes.

The pathophysiology of PPROM is intimately linked to intra-amniotic inflammation and microbial invasion of the amniotic cavity (MIAC), identified in 30%–40% of cases (4–6). Chorioamnionitis, the resulting inflammatory condition, can be diagnosed clinically (clinical chorioamnionitis, CCA) or histologically (histologic chorioamnionitis, HCA). Once membrane rupture occurs, ascending infection triggers a cascade of inflammatory responses that can progress from subclinical histologic inflammation to overt clinical chorioamnionitis. CCA relies on maternal fever plus two of the following: maternal tachycardia, fetal tachycardia, uterine tenderness, foul odor of amniotic fluid, or maternal leukocytosis. HCA, by contrast, is defined by neutrophilic infiltration of the chorion and amnion, providing objective evidence of intra-amniotic inflammation (7). HCA occurs in 40%–70% of PPROM cases, whereas CCA is identified in only 6%–10% (8). This discrepancy arises because histologic changes precede clinical manifestations, and the majority of HCA cases are subclinical, with clinical signs demonstrating poor sensitivity (as low as 26.7%) for detecting underlying inflammation (9). HCA is independently associated with increased risks of maternal and neonatal infection, preterm birth, and adverse neonatal outcomes, representing a more sensitive and objective indicator than CCA (10, 11).

Given the substantial latency period between PPROM diagnosis and delivery—particularly in expectantly managed cases before 34 weeks—there exists a critical window for early identification of intra-amniotic inflammation to guide antibiotic therapy, corticosteroid administration, and delivery timing (12, 13). However, conventional clinical indicators manifest only in advanced stages, and traditional laboratory markers lack sufficient sensitivity to reliably predict subclinical HCA, underscoring the need for alternative, objective biomarkers (9).

C-reactive protein (CRP), an acute-phase reactant synthesized by the liver in response to interleukin−6 stimulation, rises within hours of tissue injury or inflammatory insult, potentially providing an early warning signal of ongoing intra-amniotic infection (14). Newer biomarkers like procalcitonin and interleukins are promising, but they are costly and not always available. CRP, by contrast, is cheap, quick, and routinely measured in most hospitals. Previous studies have reported conflicting findings regarding the diagnostic performance of maternal serum CRP for predicting HCA in PPROM. Some studies identified CRP as an independent predictor with favorable AUC values, whereas a large prospective cohort study found CRP to be a poor overall predictor with notably low sensitivity (15%), limiting its standalone clinical utility (15–17). These inconsistencies may be attributable to differences in gestational age, diagnostic criteria, and assay methods, highlighting the need for a systematic meta-analysis to clarify the relationship between initial maternal serum CRP levels and subsequent HCA in PPROM.

Therefore, the present meta-analysis was conducted to systematically evaluate the association between maternal serum CRP concentrations at the time of initial PPROM diagnosis and the risk of subsequent HCA. By pooling data from available observational studies, we aimed to provide a robust estimate of the difference in admission CRP levels between women who developed HCA and those who did not, thereby clarifying the potential role of this non-invasive biomarker in risk stratification and early identification of intra-amniotic inflammation in PPROM. We hypothesized that higher admission CRP levels would be associated with an increased risk of subsequent HCA, given the established role of CRP as an acute-phase reactant in response to intra-amniotic infection/inflammation.

## 2 Materials and methods

### 2.1 Registration

According to the Preferred Reporting Items for Systematic Reviews and Meta-Analyses 2020 (PRISMA 2020) guidelines (18), this systematic review had its protocol registered on the International Prospective Register of Systematic Reviews (PROSPERO) under registration number CRD420261404654. To evaluate the certainty of the evidence, the Grading of Recommendations Assessment, Development and Evaluation (GRADE) framework was employed.

### 2.2 Literature search strategy

#### 2.2.1 Databases and time scope

From the inception of each database up to May 20, 2026, a comprehensive search was conducted across four electronic databases: PubMed, Scopus, Web of Science, and the Cochrane Library.

#### 2.2.2 Search terms

A combination of Medical Subject Headings (MeSH) and free-text terms was employed, with the formal search strategy finalized following multiple preliminary searches and iterative refinements. The PubMed search strategy is exemplified below: ([chorioamnionitis (Title/Abstract)] AND [PPROM[Title/Abstract] OR SPROM[Title/Abstract] OR “Preterm premature rupture of membranes”[Title/Abstract]]) AND [“c reactive protein”[Title/Abstract] OR CRP[Title/Abstract] OR hs-CRP[Title/Abstract]] AND [y_10(Filter)]. The detailed version of the full search strategy is presented in Supplementary Table S1.

### 2.3 Literature inclusion and exclusion criteria

#### 2.3.1 Inclusion criteria

Studies included in the meta-analysis must meet all of the following inclusion criteria based on the PICOS principles (19). (1) Population: Adult women (≥18 years old or referred as adult women in the article) diagnosed with PPROM were included. PPROM is defined as rupture of fetal membranes occurring before spontaneous labor at a gestational age of <37 weeks. The diagnosis is confirmed by visualization of pooling of amniotic fluid in the posterior fornix or fluid leakage from the cervical os on sterile speculum examination and a positive nitrazine test. When nitrazine testing yields inconclusive results, diagnosis is verified by a positive assay for Placental Alpha Microglobulin-1(PAMG1) or Insulin-Like Growth Factor Binding Protein-1 (IGFBP1). (2) Intervention/Exposure: Serum CRP obtained at PPROM admission. (3) Comparison: HCA-negative patients within the PPROM cohort. (4) Outcome measure: Histologically proven chorioamnionitis after delivery. HCA was diagnosed when acute inflammatory changes with neutrophilic infiltration were observed microscopically in placental chorion and fetal membranes (amnion and chorion). (5) Study design: Observational studies including cross-sectional, case-control and cohort studies.

#### 2.3.2 Exclusion criteria

Exclusion criteria: (1) multiple gestation, active labor on admission, abnormal amniotic fluid/placental location, GDM, hypertensive disorders in pregnancy, preeclampsia, antepartum hemorrhage and uterine malformation; maternal diabetes, hypertension, autoimmune, hematologic and systemic infectious diseases; fetal structural/chromosomal anomalies or intrauterine fetal demise at admission; cases with only suspected PPROM/chorioamnionitis or uncertain diagnosis. (2) Duplicate publications, case reports, conference abstracts, review articles, editorials, animal studies, *in vitro* experimental studies, studies without a control group, and non-peer-reviewed reports. (3) Some studies were published in languages other than English or Chinese. (4) Studies with insufficient raw data that could not be extracted for quantitative analysis.

### 2.4 Data extraction and quality assessment

#### 2.4.1 Data extraction

Literature screening and data extraction were conducted independently by two reviewers, following the predefined search strategy, and the results were systematically cross-checked. A standardized Excel spreadsheet was used to extract data, including first author, year of publication, study design, country, sample size, gestational age of PPROM, maternal age, CRP levels, and detection methods for CRP measurement. Any disagreements will be resolved through discussion, in cases where no agreement is achieved, a third reviewer will be consulted to arbitrate.

#### 2.4.2 Quality assessment

The methodological quality of the included observational studies was evaluated using the Newcastle-Ottawa Scale (NOS), which assesses three domains: selection of study groups, comparability, and outcome ascertainment. Based on the NOS scoring criteria, studies were classified as high quality (score ≥ 7), moderate quality (score 5–6), and low quality (score ≤ 4). Two independent researchers performed the quality assessment, and a third reviewer was consulted to resolve any disagreements. All studies enrolled in the final meta-synthesis were rated as moderate to high quality with an overall low risk of bias.

#### 2.4.3 Grading the quality of evidence

The certainty of evidence was assessed using the Grading of Recommendations Assessment, Development and Evaluation (GRADE) framework across five domains (risk of bias, inconsistency, indirectness, imprecision, and publication bias). For observational studies (cross-sectional and case-control), the baseline certainty was rated as low and subsequently adjusted based on domain-specific factors. The final Summary of Findings (SoF) table was generated using GRADEpro Guideline Development Tool (GDT) software.

### 2.5 Statistical analysis

All statistical analyses were performed using R software (version 4.6.0). Serum CRP was naturally log-transformed due to right-skewed distribution. For studies reporting median and interquartile ranges (IQRs), log-transformed mean and standard deviation (SD) were estimated using Wan's method (20). Meta-analysis was performed on log-transformed CRP using mean difference (MD). Results were back-transformed to geometric mean ratio for clinical interpretation.

Between-study heterogeneity was evaluated using the *I*^2^ statistic and Cochran's *Q* test. According to the Cochrane Handbook, heterogeneity was defined as negligible (*I*^2^ < 25%), low (*I*^2^ = 25%–50%), moderate(*I*^2^ = 50%–75%), and substantial(*I*^2^ = 75%–100%). Pooled MD with 95% confidence intervals (CIs) were calculated using a random-effects model (REM) when *I*^2^ exceeded 50%; otherwise, a fixed-effects model (FEM) was applied.

Subgroup analysis was conducted to explore potential sources of heterogeneity. Sensitivity analysis was performed by sequentially omitting one individual study at a time to evaluate the robustness of the pooled results. Publication bias targeting the primary pooled effect was assessed using funnel plot visualization, alongside three complementary quantitative methods: Egger's linear regression test, Begg's rank correlation test, and trim-and-fill adjustment.

Meta-regression analysis was carried out using the metareg() function from the meta package in R to explore sources of between-study heterogeneity. Predefined moderators included mean age of patients with HCA, gestation age at PPROM, sample size, and publication year. For each moderator, regression coefficients, standard errors, z-values, *P*-values, and 95% CIs were calculated. The explanatory capacity of each moderator was quantified by the proportion of between-study variance explained (*R*^2^) (21).

## 3 Results

### 3.1 Literature screening results

In accordance with the PRISMA 2020 guidelines, a total of 388 records were initially retrieved from four databases, including 169 from Scopus, 164 from Web of Science, 49 from PubMed, and 6 from the Cochrane Library, with no additional records identified through other sources. After duplicate removal using Covidence software (*n* = 161), 227 unique records proceeded to title–abstract evaluation. Then 135 records were excluded, leaving 92 studies for full-text assessment. In the full-text screening, 81 studies were excluded for the following reasons: wrong data (1), no full text (*n* = 5), duplicate results (*n* = 4), Paediatric population (*n* = 1), not English or Chinese (*n* = 6), wrong patient population (*n* = 1), failure to get indicated data (*n* = 63). Ultimately, 11 eligible studies were included in the final meta-analysis. The entire literature selection process is presented in the PRISMA flow diagram (Figure 1).

**Figure 1:**
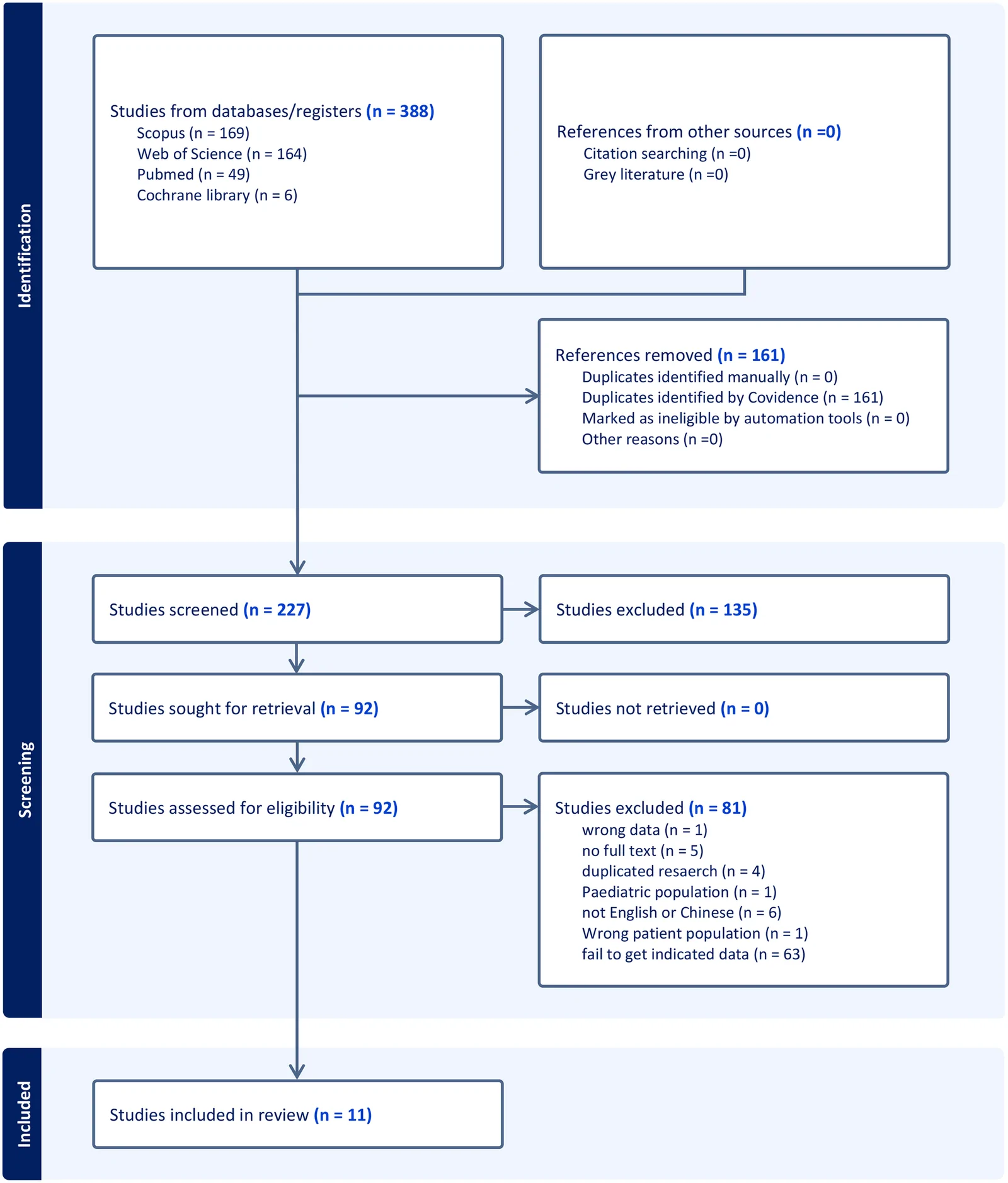
PRISMA flow diagram of literature screening.

### 3.2 Basic characteristics of included studies

The methodological quality of the initially included 11 studies was assessed using the NOS. One study scored 5 points indicating poor methodological quality and was therefore excluded (Supplementary Table S2). Finally, 10 eligible studies published between 2013 and 2026 were included in the meta-analysis. Among all participants, 861 were in the HCA group and 930 in the non-HCA group. The sample size per study ranged from 12 to 261participants. The included studies covered three geographical regions: East Asia, Europe, North America (Table 1). All included studies had NOS scores of = 7, indicating high methodological quality (Table 2).

**Table 1:** Basic characteristics of the included literature.

Study	Year	Study design	Country	Sample size (HCA/Control)	HCA Gestational age of PPROM (Weeks)	Control Gestational age of PPROM (Weeks)	HCA age (year)	Control age (year)	HCA CRP (mg/dL)	Control CRP (mg/dL)	Detection method
Cho 2024 (27)	2024	retrospective cohort study	Korea	88/84	29.4 ± 3.0	31.3 ± 2.7	32.0 ± 3.6	31.2 ± 4.2	1.29 ± 1.70	0.49 ± 0.62	turbidimetric immunoassay
Çakar 2016 (28)	2016	Prospective cohort study	Turkey	41/12	25 (23–27)	25.5 (24–27.8)	28.3 ± 6.1	29.7 ± 7.2	1.40 (0.45–2.30)	0.95 (0.35–2.18)	immunonephelometry
Stepan 2016 (15)	2016	retrospective cohort study	Czech Republic	116/141	32.57 (31.14–35)	33.14 (31.71–35.14)	32 (28–35)	30 (27–34)	0.64 (0.35–0.99)	0.45 (0.2–0.85)	immunoturbidimetric analysis
LeRay 2015 (29)	2015	prospective study	France	54/67	NA	NA	31.29 ± 5.27	29.73 ± 6.08	1.269 ± 1.846	0.772 ± 0.842	NA
Galletta 2023 (30)	2023	retrospective cohort study	Brazil	72/223	30.76 ± 4.539	33.48 ± 3.784	NA	NA	2.017 ± 2.861	1.281 ± 1.559	NA
Cho 2017 (31)	2017	retrospective cohort study	Korea	62/87	31.57（20–36.43）	33.29（20.43–36.86）	32.8 ± 3.3	33.4 ± 3.4	7.5 (0.6–65.2)	3.3 (0.6–32.0)	nephelometry
Kong 2023 (32)	2023	retrospective cohort study	China	93/113	33 (30–34)	34 (32–35)	32 (29–35)	33 (29–35)	0.952 (0.530–1.760)	0.628 (0.452–0.94)	nephelometry
Xie 2015 (33)	2015	retrospective cohort study	China	261/110	30.9 ± 1.6	31.7 ± 1.3	27.6 ± 4.8	28.2 ± 4.8	0.92 ± 0.71	0.77 ± 0.69	NA
Hu 2026 (34)	2026	retrospective cohort study	China	53/65	32.42 ± 2.13	32.82 ± 2.31	29.72 ± 3.56	30.02 ± 3.58	1.565 (1.169, 2.079)	0.834 (0.543, 1.253)	NA
Çekmez 2013 (35)	2013	retrospective cohort study	Turkey	21/28	30.05 ± 1.8	31 ± 2.2	25.2 ± 2.87	25.5 ± 3.6	1.82 ± 0.31	0.81 ± 0.37	Immunoturbidimetric assay

Data are presented as mean ± SD or median (interquartile ranges), as described in the original article. HCA, histologic chorioamnionitis; CRP, C-reactive protein; PPROM, preterm premature rupture of membranes.

**Table 2:** The Newcastle-Ottawa scale served to assess the risk of bias in the included studies.

Study	Selection				Comparability		Exposure			Summary	
	Representativeness of the exposed cohort	Selection of the nonexposed cohort	Ascertainment of exposure	Demonstration that outcome of interest was not present at start of study	Matched in design	Adjusted in analyses	Assessment of outcome	Adequate follow up period for outcome of interest	Adequacy of follow up of cohorts	Total point	Risk of bias
Cho 2024 (27)	1	1	1	1	0	0	1	1	1	7	low
Çakar 2016 (28)	1	1	1	1	0	0	1	1	1	7	low
Stepan 2016 (15)	1	1	1	1	0	0	1	1	1	7	low
LeRay 2015 (29)	1	1	1	1	0	0	1	1	1	7	low
Galletta 2023 (30)	1	1	1	1	0	0	1	1	1	7	low
Cho 2017 (31)	1	1	1	1	0	0	1	1	1	7	low
Kong 2023 (32)	1	1	1	1	0	0	1	1	1	7	low
Xie 2015 (33)	1	1	1	1	0	0	1	1	1	7	low
Hu 2026 (34)	1	1	1	1	0	0	1	1	1	7	low
Çekmez 2013 (35)	1	1	1	1	0	0	1	1	1	7	low

### 3.3 Meta-analysis of CRP level between HCA and non-HCA group

Pooled analysis of 10 eligible studies was performed to compare the log-transformed CRP levels between PPROM patients with HCA and without HCA. Significant between-study heterogeneity was observed (*χ*^2^ = 72.90, *P* < 0.001, *I*^2^ = 87.7%), therefore the random-effects model was adopted. The pooled MD was 0.439 (95%CI: 0.179–0.700), demonstrating that serum log-transformed CRP levels were significantly higher in HCA group than in non-HCA group (Figure 2).

**Figure 2:**
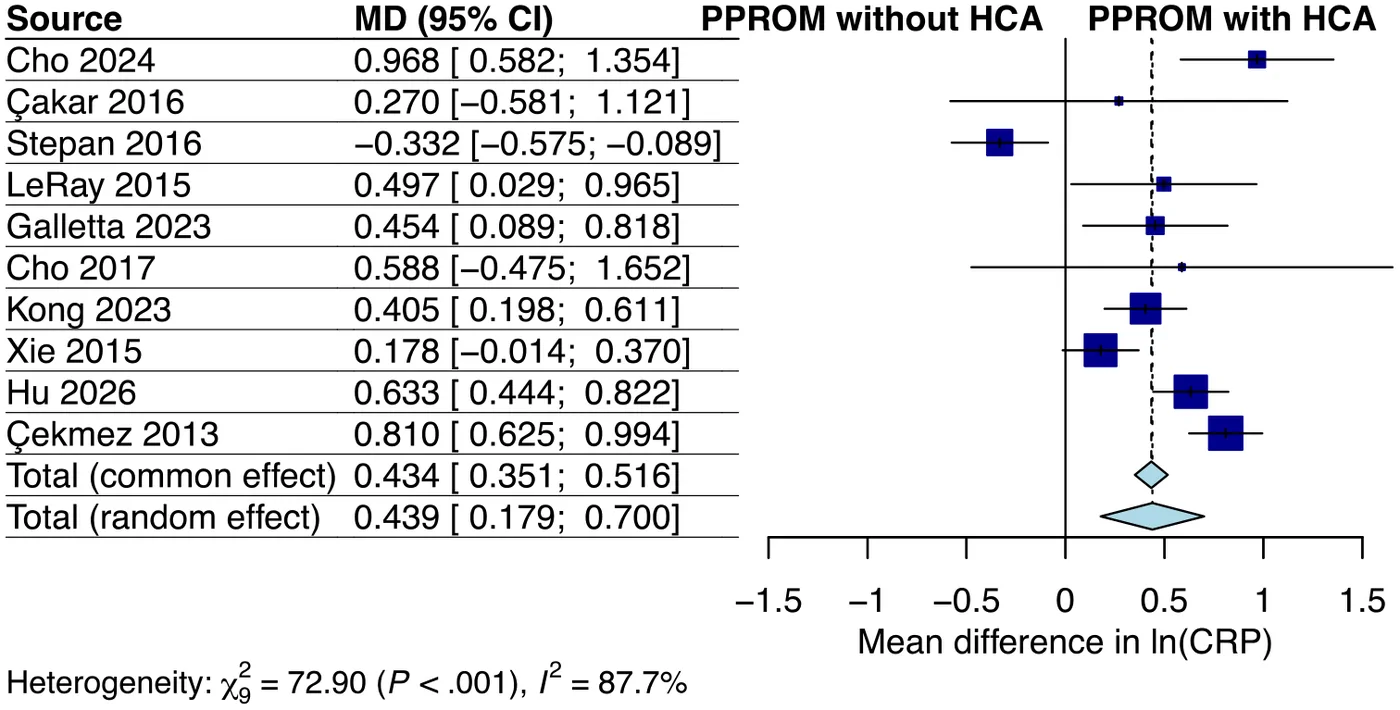
Forest plot for pooled mean difference of natural log-transformed CRP between PPROM with and without HCA.

### 3.4 Sensitivity analysis

We additionally included the low-quality study (22), which achieved an NOS score of 5, and repeated the pooled meta-analysis across all 11 eligible cohort studies. The pooled random-effects MD was 0.443 (95%CI: 0.179–0.707), highly consistent with the primary outcome of MD = 0.439 (95%CI: 0.179–0.700). This study account for merely 0.2% to the total weight, with an extremely wide confidence interval crossing zero, thus exerting no substantial impact on the pooled effect (Supplementary Figure 1A). Furthermore, three sensitivity analyses were performed to verify result robustness. After excluding the only negative study (Stepan 2016), pooled MD was 0.543 (95% CI: 0.348–0.738), and heterogeneity decreased from 87.7% to 73.2%, demonstrating that this study was the major contributor to overall heterogeneity (Supplementary Figure 1B). Excluding five studies with median and IQRs-derived data yielded a pooled MD of 0.575 (95% CI: 0.246–0.904) (Supplementary Figure 1C). Leave-one-out analysis showed pooled MD ranged from 0.379 to 0.543, with all 95% CIs excluding zero (Figure 3). Collectively, multi-angle sensitivity tests confirmed the robustness of our core finding that log-transformed CRP was significantly higher in the HCA group than in the non-HCA.

**Figure 3:**
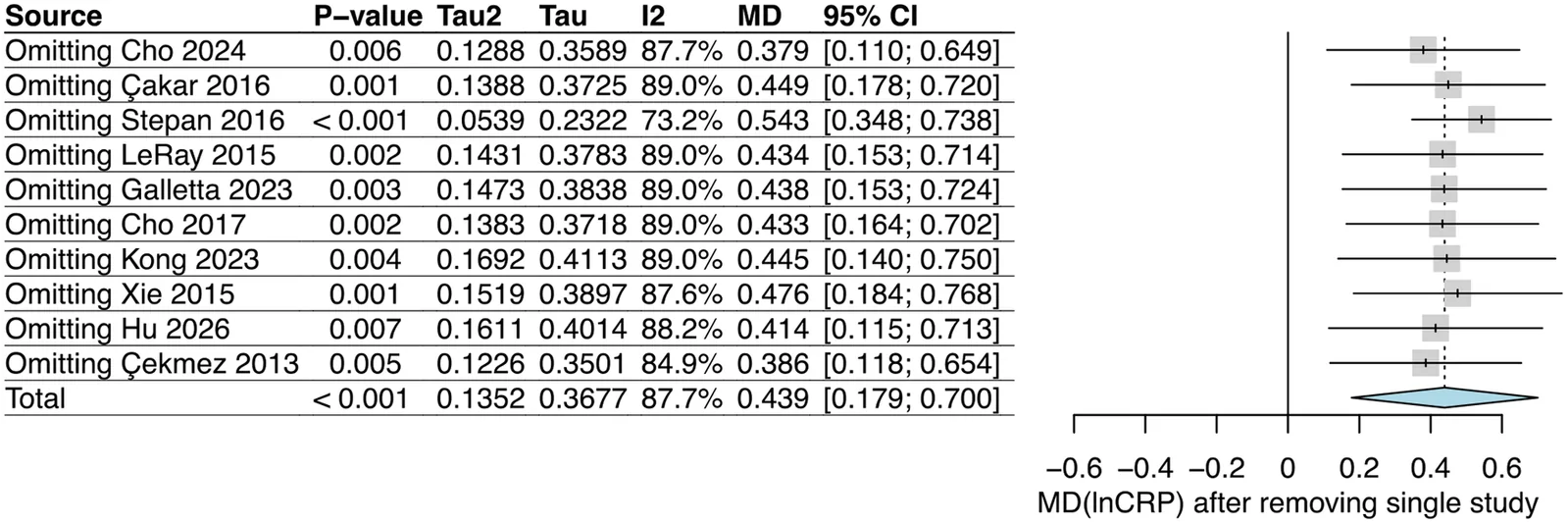
Leave-one-out sensitivity analysis.

### 3.5 Subgroup analysis

Predefined subgroup analyses were performed based on maternal age (Figure 4A), publication year (Figure 4B), study region (Figure 4C), CRP detection assays (Figure 4D), sample size (Figure 4E), and gestational age at PPROM onset (Figure 4F). Between-subgroup comparisons revealed a borderline significant difference only for sample size (*P* = 0.05), while no significant differences were observed for all other factors (all *P* > 0.05). To reduce conversion bias arising from the right-skewed distribution of gestational age, we also conducted sequential sensitivity analyses using Luo's weighted formula (Supplementary Figure 2A), and grouping by raw medians (Supplementary Figure 2B) besides long-normal estimation (Figure 4F). The pooled MD, heterogeneity statistic, and *P*-values for subgroup comparisons remained nearly identical across analyses. Although subgroup interaction tests were non-significant for most variables, descriptive patterns suggested larger CRP–HCA associations in early PPROM (<32 weeks), younger maternal age, smaller studies, and recent publications. However, these apparent differences lacked statistical confirmation, likely reflecting limited power for formal interaction testing.

**Figure 4:**
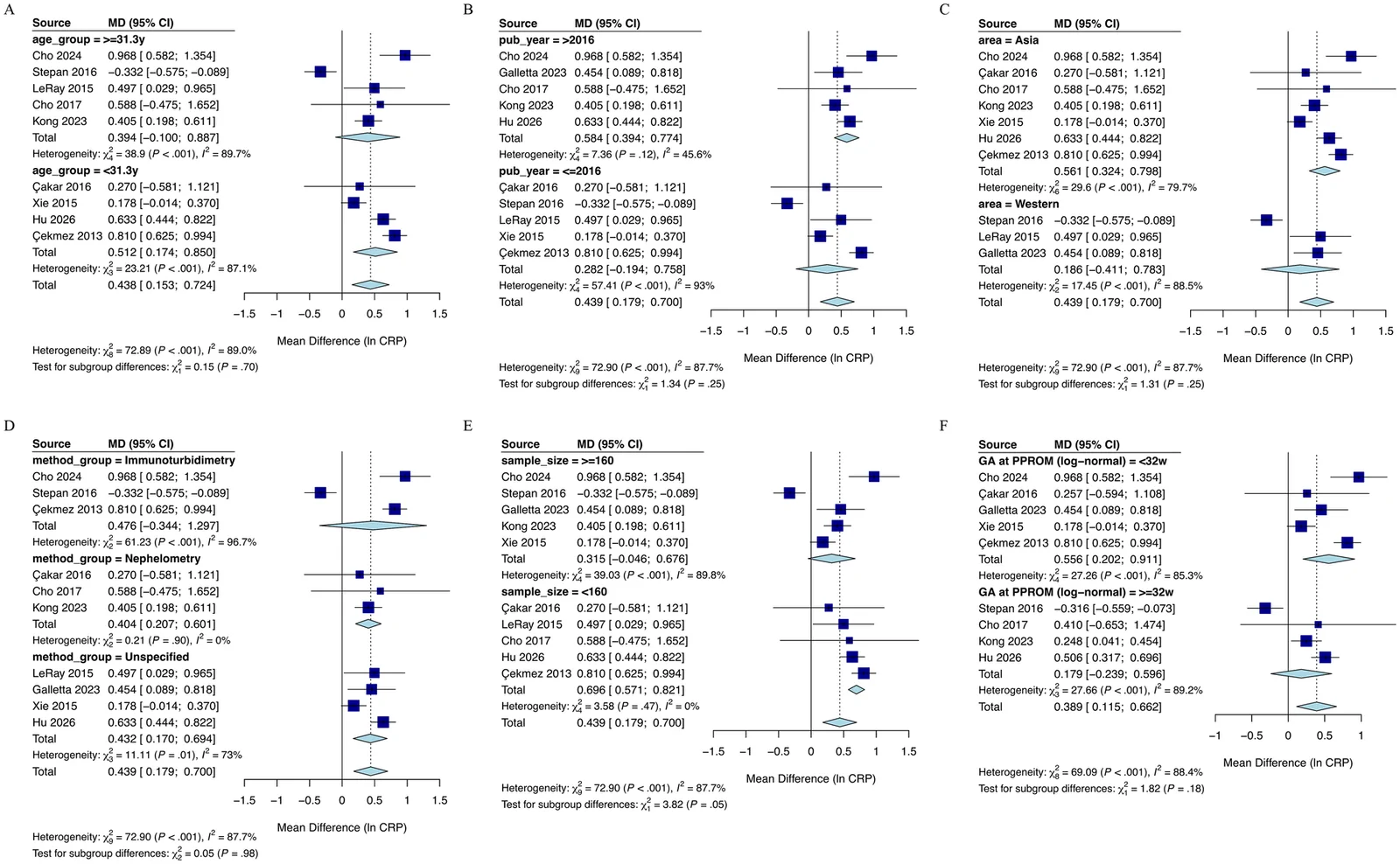
Subgroup analyses of the MD in serum CRP levels between PPROM patients with and without subsequent HCA. **(A)** Maternal age. **(B)** Publication year. **(C)** Geographic region. **(D)** CRP detection method. **(E)** Study sample size. **(F)** Gestational age at PPROM onset.

### 3.6 Meta regression

To explore potential moderators driving between-study heterogeneity, univariate meta-regression analyses were performed. Sample size showed a borderline inverse moderating effect on the MD of log-transformed CRP (*β* = −0.002, *P* = 0.0636), which explained 31.19% of cross-study variance (Figure 5A). No significant moderating effects were observed for gestational age at PPROM onset (*β* = −0.069, *P* = 0.3385, *R*^2^ = 8.99%, Figure 5B), publication year (*β* = −0.0284, *P* = 0.6453, *R*^2^ = 0%, Figure 5C), or mean maternal age (*β* = 0.0253, *P* = 0.4163, *R*^2^ = 0%, Figure 5D). Collectively, among the covariates examined, only sample size showed a trend toward explaining heterogeneity, although the association did not reach statistical significance.

**Figure 5:**
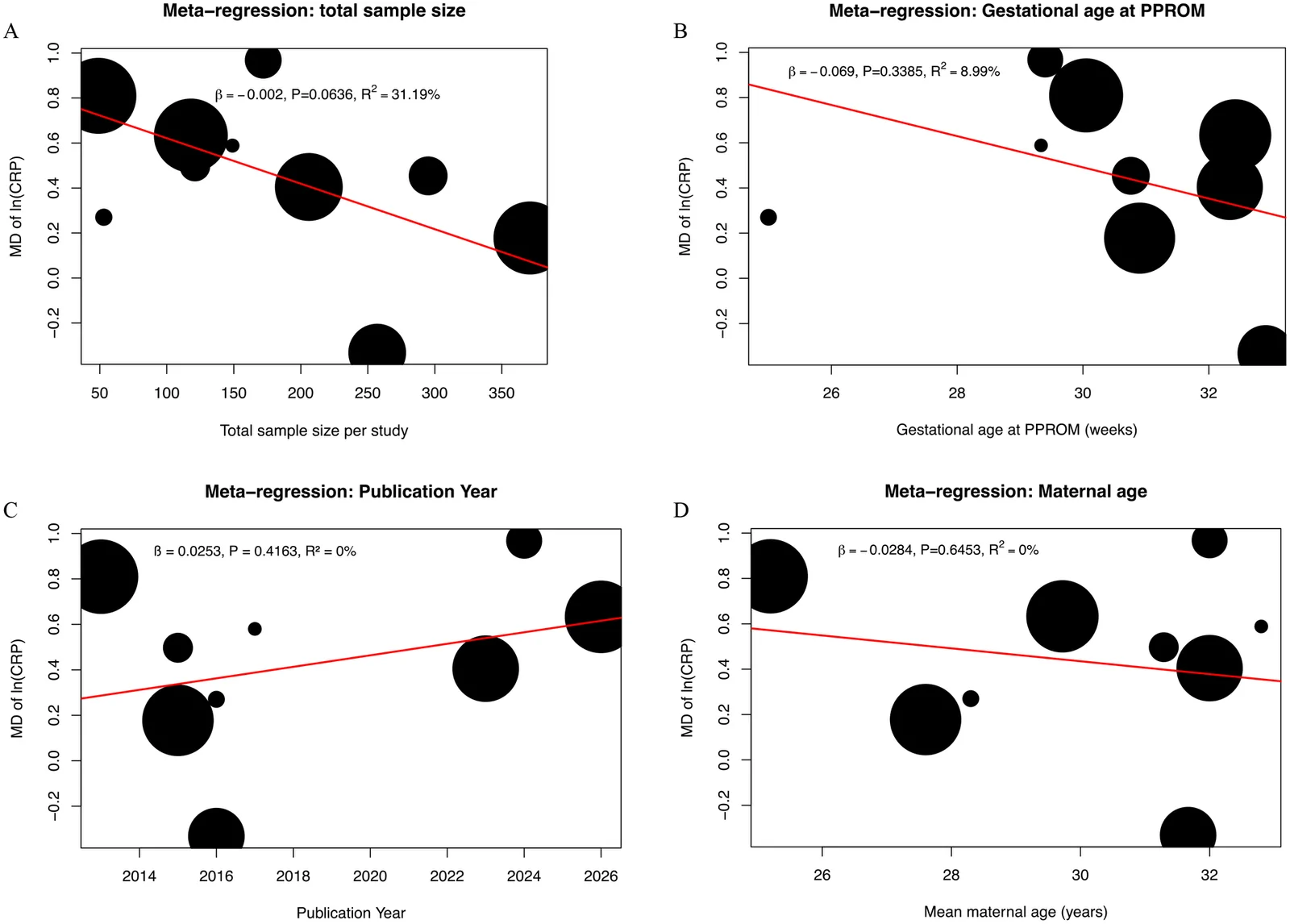
Meta-regression analyses exploring the sources of between-study heterogeneity for the MD of log-transformed CRP levels. **(A)** Total sample size per study. **(B)** Gestational age at PPROM onset. **(C)** Publication year. **(D)** Mean maternal age.

### 3.7 Publication bias

Given the limited number of included studies, publication bias was assessed using multiple complementary methods. Funnel plot, Egger's test, and Begg's test were used to assess publication bias. With only 10 included studies, these statistical assessments had limited power to detect small-study asymmetry. No obvious visual asymmetry was detected in the funnel plot (Figure 6), and neither Egger's test (*P* = 0.088) nor Begg's test (*P* = 1.000) indicated statistically significant publication bias (Supplementary Table S3). Trim-and-fill analysis was further performed to assess the robustness of the pooled effect against potential publication bias. After imputing 3 hypothetical missing small-sample studies on the left side of the funnel plot (Supplementary Table S3), the pooled MD decreased from 0.439 to 0.249 (95% CI: −0.018 to 0.517, *P* = 0.068). Although the positive direction of the effect remained consistent, the pooled effect lost statistical significance, suggesting that the original significant finding may be at least partially attributable to publication bias, and should be interpreted with caution.

**Figure 6:**
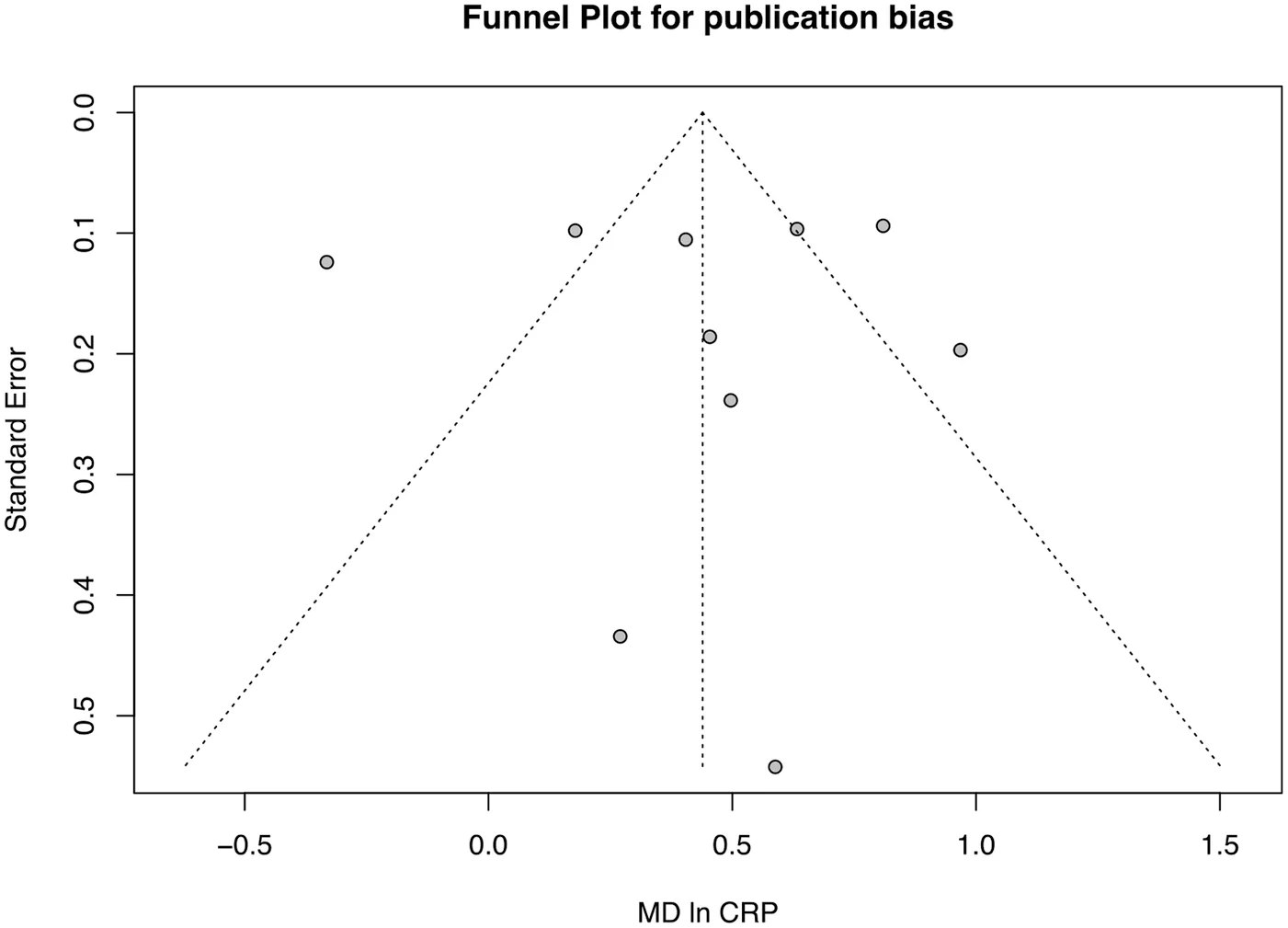
Funnel plot for assessing publication bias.

### 3.8 GRADE evidence quality assessment

The GRADE framework was used to evaluate evidence certainty. Given that all included studies were observational (cohort studies), the initial certainty was rated as low. In view of serious risk of bias (moderate methodological limitations in the majority of studies) and serious inconsistency (marked heterogeneity, *I*^2^ = 87.7%), we downgraded the evidence by two levels, yielding a final rating of very low (Supplementary Table S4).

## 4 Discussion

Existing meta-analyses focusing on CRP in PPROM patient primarily evaluated the diagnostic performance of CRP for HCA (23–26). However, these studies fail to address a clinically meaningful prospective question: whether baseline CRP concentration measured at the time of PPROM can predict the subsequent risk of incident HCA. Targeting the core clinical demand for risk prediction in PPROM management, the present study shifts the research perspective and conducts a meta-analysis to explore the prospective association between baseline CRP concentrations at PPROM presentation and subsequent development of HCA.

Data pooled from 10 eligible cohort studies demonstrated higher admission CRP levels among PPROM patients who developed subsequent HCA. The pooled MD for log-transformed CRP was 0.439 (95% CI: 0.179–0.700), corresponding to a geometric mean ratio of 1.551 (95% CI: 1.196–2.014). Despite the significant association, the modest effect size and wide confidence interval suggest that CRP alone may not suffice for individual risk stratification, but could serve as a component of a multivariable prediction model.

Publication bias warrants particular attention when interpreting our findings. We found inconsistent signals across publication bias assessments. Visual funnel inspection and rank-based correlation tests did not signal meaningful asymmetry, yet trim-and-fill imputation implied three potentially missing studies. This discrepancy is likely attributable to the limited number of included studies (*k* = 10), which reduces the ability of statistical tests to reliably detect subtle funnel asymmetry. The attenuation of the pooled effect to non-significance after imputation underscores that the original significant association may be partially driven by selective publication of positive results. Additionally, our restriction to published literature without grey literature retrieval may have further amplified this risk. Therefore, despite the non-significant Egger's test, the possibility of publication bias cannot be excluded, and the pooled estimates should be interpreted with this limitation in mind.

Substantial heterogeneity was observed in the overall pooled analysis (*I*^2^ = 87.7%). To identify potential sources, we performed subgroup analyses and meta-regression. Subgroup analyses suggested several factors that may partially explain the observed inconsistency, though none emerged as a predominant source. Subgroup stratification by sample size showed that small cohorts presented uniform positive CRP–HCA associations with *I*^2^ = 0%, while larger studies showed substantial heterogeneity. The apparent consistency within small cohorts likely reflects limited statistical power to detect between-study variance rather than genuine uniformity, as small studies are more susceptible to extreme effect estimates due to random sampling error. Notably, a similar pattern was observed in the exploratory subgroup analysis by CRP assay method, where immunoturbidimetry-based studies also showed *I*^2^ = 0%. However, as only one study used nephelometry and the test for subgroup differences was not significant, this finding remains exploratory. When stratified by publication year, studies published after 2016 displayed much lower heterogeneity than earlier work. Despite the non-significant subgroup difference test confirming publication year was not a formal moderator of cross-study inconsistency, we reasonably infer that progressive standardization of obstetric diagnostic and laboratory protocols in recent years may partially reduce study-level variation. Considerable residual heterogeneity remained after subgroup and meta-regression analyses. Although we adopted a random-effects model to accommodate between-study variance, the pooled effect merely reflects an averaged association derived from heterogeneous clinical, laboratory and methodological settings rather than a universal estimate suitable for all PPROM populations. Given the substantial residual inconsistency across studies, the overall result requires cautious interpretation.

Multiple clinical and methodological factors may explain the residual heterogeneity. Baseline CRP concentrations varied among individuals due to differences in maternal health status and pre-pregnancy inflammatory levels. Methodological discrepancies across studies also likely contributed: CRP measurement techniques differed, with some studies employing nephelometry and others immunoturbidimetry. Although most studies used the same histopathological definition of HCA, the lack of standardized grading systems or uniform neutrophil infiltration thresholds may still have introduced diagnostic variability. Furthermore, the interval from PPROM onset to venous blood draw lacked uniform standards across trials, and this variable sampling window directly altered measured CRP concentrations. Several studies only reported median and interquartile range, and data transformation introduced methodological bias. Beyond these methodological differences, clinical heterogeneity across study populations also played a role. Notably, sensitivity analysis confirmed Stepan et al. (2016) as a key contribution to cross-study inconsistency, as removing this cohort reduced heterogeneity from 87.7% to 73.2%. Unlike other studies comparing PPROM patients with HCA-positive and HCA-negative, this study used a more detailed stratification, including patients with HCA complicated by MIAC, isolated HCA, isolated MIAC, and uninfected controls, generating an opposing overall effect that constituted a major source of heterogeneity. Other unmeasured confounders—such as maternal body mass index, latency period from PPROM to delivery, and antenatal antibiotic or corticosteroid administration—may have further influenced CRP levels across studies but could not be quantified in our meta-regression. These findings indicate that future studies should standardize CRP assay protocols, blood sampling timing, and HCA diagnostic criteria, in addition to grouping criteria and adjustment for concomitant MIAC, to reduce clinical and methodological heterogeneity when investigating the association between HCA and CRP.

Our findings should be interpreted in the context of several limitations. First, our literature search was restricted to English and Chinese publications and to published works without grey literature retrieval. Relevant studies in other languages—particularly those reporting negative or null findings—may have been missed, potentially overestimating the pooled effect. The absence of grey literature may have further amplified publication bias. Second, the number of included studies was relatively small (*k* = 10), with limited sample sizes, which compromised the statistical power of publication bias tests and subgroup analyses. While Egger's test did not reveal significant asymmetry, this does not exclude publication bias, as such tests are underpowered when fewer than 10 studies are included. Third, considerable unexplained between-study heterogeneity remained despite our subgroup and meta-regression analyses. While we have discussed several potential sources of heterogeneity, the observational nature of the included studies precludes adjustment for all confounders, and individual patient-level data were not available to formally quantify the independent contribution of each factor. The residual inconsistency limits the precision of our pooled estimate and warrants cautious interpretation. Fourth, CRP is a non-specific inflammatory marker, and its predictive performance may be influenced by concurrent infections or inflammatory conditions unrelated to intra-amniotic inflammation. Fifth, the statistical association we observed should not be equated with diagnostic utility. A significant group-level mean difference does not mean that CRP can reliably classify individual patients as HCA-positive or HCA-negative. The small effect size further argues against using a single CRP measurement as a standalone diagnostic test for HCA. Sixth, from a clinical standpoint, admission CRP has limited value as a standalone marker. It may offer useful information when interpreted alongside other clinical parameters or incorporated into a multivariable predictive model. But it should never replace clinical judgment. Seventh, the GRADE certainty of evidence was rated as very low, reflecting serious risk of bias and inconsistency across the included observational studies, further underscoring the exploratory nature of our findings.

## 5 Conclusion

Current evidence indicates that elevated serum CRP at admission is directionally associated with subsequent HCA in PPROM patients. However, given the modest effect size, substantial heterogeneity, potential publication bias, and very low certainty of evidence, CRP should not be interpreted as a reliable standalone predictor. Rather, it may serve as an auxiliary biomarker for early risk stratification within a multilevel assessment framework. Elevated CRP may prompt closer monitoring and earlier consideration of delivery, while normal CRP cannot exclude HCA owing to its low sensitivity. Future research should focus on dynamic biomarker trajectories and multi-marker panels to advance precision management of PPROM.
