# Supplementary material for: Association of admission C-reactive protein levels and histological chorioamnionitis in patients with preterm premature rupture of membranes: a systematic review and meta-analysis
Source: Front Reprod Health. 2026 Sep 28;8:1912720. doi: 10.3389/frph.2026.1912720 (PMC13655316; doi:10.3389/frph.2026.1912720)
Supplement: Supplementary file 1 [file Table1.docx]

| **Supplementary Materials Table S1. Search strategy** | |  |  |  |
| --- | --- | --- | --- | --- |
| Database | Search strategy | | Results | Time |
| Scopus | ( TITLE-ABS-KEY ( chorioamnionitis ) AND TITLE-ABS-KEY ( PPROM OR SPROM OR "Preterm premature rupture of membranes" ) AND TITLE-ABS-KEY ( "c reactive protein" OR CRP OR hs-CRP ) ) AND ( LIMIT-TO ( DOCTYPE , "ar" ) ) | | 169 | May 20,2026 |
| PubMed | ((chorioamnionitis[Title/Abstract]) AND (PPROM[Title/Abstract] OR SPROM[Title/Abstract] OR "Preterm premature rupture of membranes"[Title/Abstract])) AND ("c reactive protein"[Title/Abstract] OR CRP[Title/Abstract] OR hs-CRP[Title/Abstract]) AND (y_10[Filter]) | | 49 | May 20,2026 |
| Cochrane  Library | (chorioamnionitis):ti,ab,kw AND (PPROM OR SPROM OR "Preterm premature rupture of membranes"):ti,ab,kw AND ("c reactive protein" OR CRP OR hs-CRP):ti,ab,kw" | | 6 | May 20,2026 |
| Web of Science | chorioamnionitis (Topic) AND PPROM OR SPROM OR "Preterm premature rupture of membranes" (Topic) AND "c reactive protein" OR CRP OR hs-CRP (Topic) and Article (Document Types) | | 164 | May 20,2026 |

Supplementary Material

**Supplementary Materials Table S2. NOS**

| Study | Selection | | | | Comparability | | Exposure | | | Summary | |
| --- | --- | --- | --- | --- | --- | --- | --- | --- | --- | --- | --- |
|  | Adequate  Case  definition | Case representativeness | Control selection | Control definition | Matched in design | Adjusted in analyses | Exposure ascertainment | Same method of ascertainment for cases and controls | non-response rate for both groups | Total point | Risk of bias |
| Soydinç 2012 | 1 | 1 | 0 | 1 | 0 | 0 | 1 | 1 | 0 | 5 | Moderate |

| **Supplementary Materials Table S3. Publication_Bias_Detection_Summary** | | | |
| --- | --- | --- | --- |
| Test_Method | Statistical_Index | P_Value | Interpretation |
| Egger's Regression Test | Intercept = 0.408 (SE = 0.210) | 0.088 | No significant publication bias exists |
| Begg's Rank Correlation Test | Z = 0 | 1.00 | No significant publication bias exists |
| Trim-and-Fill Imputed Studies | 3 missing studies imputed | N/A | 3 studies imputed on the left side; pooled effect attenuated but did not reverse |

| **Supplementary Materials Table S4. GRADE for quality of evidence profile** | | | | | | | | | | | | |
| --- | --- | --- | --- | --- | --- | --- | --- | --- | --- | --- | --- | --- |
| Outcomes | Study design | | Number of studies(patients) | | Risk of bias | Inconsistency | Indirectness | Imprecision | Other considerations | Relative effect (95% CI) | Certainty |  |
| CRP (log-transformed) | Observational Studies | 10(1791) | | Serious^a^ | | Very serious^b^ | Not Serious | Not Serious | None | MD 0.439 (95% CI 0.179 to 0.700) | ⊕⊝⊝⊝  Very Low |  |
|  | Note: a, all included studies adopted an observational cohort design without blinding of laboratory testers or outcome assessors, with unadjusted or incompletely adjusted confounding factors; this factor downgraded evidence certainty by one level. b, marked inter-study heterogeneity was observed (*I*² = 87.7 %). Subgroup and meta-regression analyses only partially explained cross-study variance, with prominent residual heterogeneity remaining. This very serious inconsistency led to a two-level downgrade of the overall evidence certainty. CI, confidence interval; MD**,** mean difference; GRADE, Grading of Recommendations Assessment, Development and Evaluations. | | | | | | | | | | |  |

**Supplementary Figure 1.** Primary and sensitivity analyses of the MD in serum CRP (log-transformed) between PPROM patients with and without HCA.

A. Primary meta-analysis forest plot including all eligible studies.

B. Sensitivity analysis excluding the outlying study (Stepan 2016).

C. Sensitivity analysis excluding studies reporting median and interquartile range (IQR) data.

**Supplementary Figure 2.** Subgroup analysis of the association between serum log-transformed CRP levels and subsequent HCA, stratified by gestational age (GA) at PPROM onset using two different grouping methods.

A. Luo method for gestational age categorization.

B. The raw median gestational age of the included studies as the cut-off
